# Supplementary material for: The Coli Toolkit (CTK): An Extension of the Modular Yeast Toolkit for Use in E. coli
Source: ACS Synth Biol. 2026 Jan 25;15(2):462–71. doi: 10.1021/acssynbio.5c00489 (PMC12930495; doi:10.1021/acssynbio.5c00489)
Supplement: Supplementary file 1 [file sb5c00489_si_001.pdf]

Supplementary Information

The Coli Toolkit (CTK): An extension of the modular Yeast Toolkit for use in *E. coli*

Jacob Mejlsted[1,2,3], Erik Kubaczka[1,3], Sebastian Wirth[1,3], and Heinz Koeppl[1,3]*

1. Centre for Synthetic Biology, TU Darmstadt, Darmstadt 64283, Germany

2. Graduate School Life Science Engineering, TU Darmstadt, Darmstadt 64283, Germany

3. Department of Electrical Engineering and Information Technology, TU Darmstadt, Darmstadt 64283, Germany

*Corresponding author: heinz.koeppl@tu-darmstadt.de

Supporting Tables and Figures

Supplementary Table S1	List of Coli Toolkit plasmids
Supplementary Table S2	List of plasmids made for characterization results
Supplementary Table S3	Constitutive promoter expression levels
Supplementary Table S4	Inducible promoters dose-response parameters
Supplementary Table S5	NOT gate response function parameters
Supplementary Figure S1	Ligase fidelity overview of the 4-base pair overhangs employed in the CTK
Supplementary Figure S2	Distributions of input promoters
Supplementary Figure S3	Distributions of NOT gates
Supplementary Figure S4	Response functions of NOT gates.

Supporting Text

Supplementary Text	Benchmarking clustering algorithm

Supplementary Table S5	Benchmark of set 1, the CTK parts
Supplementary Table S6	Benchmark of set 2, the parts required for the 2-bit hashing function
Supplementary Table S7	Benchmark of set 3, RcsAB promoters

Supplementary Table S1: List of Coli Toolkit plasmids

Plasmid	*Type*	*Name*	*Function*	*Resistance*	*Part source*
pCTK001	2ab	PAmeR	Promoter	CamR	[1]
pCTK002	2ab	PAmtR	Promoter	CamR	[1]
pCTK003	2ab	PBAD	Promoter	CamR	[1]
pCTK004	2ab	PBetI	Promoter	CamR	[1]
pCTK005	2ab	PBM3R1	Promoter	CamR	[1]
pCTK006	2ab	PHlyIIR	Promoter	CamR	[1]
pCTK007	2ab	PIcaRA	Promoter	CamR	[1]
pCTK008	2ab	PLitR	Promoter	CamR	[1]
pCTK009	2ab	PLmrA	Promoter	CamR	[1]
pCTK010	2ab	PPhlF	Promoter	CamR	[1]
pCTK011	2ab	PPsrA	Promoter	CamR	[1]
pCTK012	2ab	PQacR	Promoter	CamR	[1]
pCTK013	2ab	PSrpR	Promoter	CamR	[1]
pCTK014	2ab	PTac	Promoter	CamR	[1]
pCTK015	2ab	PTet	Promoter	CamR	[1]
pCTK016	2ab	PLacI	Promoter	CamR	[1]
pCTK017	2a	PAmtR	Promoter	CamR	[1]
pCTK018	2a	PBAD	Promoter	CamR	[1]
pCTK019	2a	PHlyIIR	Promoter	CamR	[1]
pCTK020	2a	PPhlF	Promoter	CamR	[1]
pCTK021	2a	PSrpR	Promoter	CamR	[1]
pCTK022	2a	PTac	Promoter	CamR	[1]
pCTK023	2a	PTet	Promoter	CamR	[1]
pCTK024	2b	PAmeR	Promoter	CamR	[1]
pCTK025	2b	PAmtR	Promoter	CamR	[1]
pCTK026	2b	PBetI	Promoter	CamR	[1]
pCTK027	2b	PhlyIIR	Promoter	CamR	[1]
pCTK028	2b	PTet	Promoter	CamR	[1]
pCTK029	2ab	J23101	Constitutive promoter	CamR	[2]
pCTK030	2ab	J23102	Constitutive promoter	CamR	[2]
pCTK031	2ab	J23103	Constitutive promoter	CamR	[2]
pCTK032	2ab	J23104	Constitutive promoter	CamR	[2]
pCTK033	2ab	J23105	Constitutive promoter	CamR	[2]
pCTK034	2ab	J23106	Constitutive promoter	CamR	[2]
pCTK035	2ab	J23107	Constitutive promoter	CamR	[2]
pCTK036	2ab	J23108	Constitutive promoter	CamR	[2]
pCTK037	2ab	J23109	Constitutive promoter	CamR	[2]
pCTK038	2ab	J23110	Constitutive promoter	CamR	[2]
pCTK039	2ab	J23111	Constitutive promoter	CamR	[2]
pCTK040	2ab	J23113	Constitutive promoter	CamR	[2]
pCTK041	2ab	J23115	Constitutive promoter	CamR	[2]
pCTK042	2ab	J23116	Constitutive promoter	CamR	[2]
pCTK043	2ab	J23117	Constitutive promoter	CamR	[2]
pCTK044	2ab	J23118	Constitutive promoter	CamR	[2]
pCTK045	2ab	J23119	Constitutive promoter	CamR	[2]
pCTK046	2c	BydvJ	Ribozyme insulator	CamR	[1]
pCTK047	2c	ElvJ	Ribozyme insulator	CamR	[1]
pCTK048	2c	PlmJ	Ribozyme insulator	CamR	[1]
pCTK049	2c	RiboJ	Ribozyme insulator	CamR	[1]
pCTK050	2c	RiboJ10	Ribozyme insulator	CamR	[1]

pCTK051	2c	RiboJ51	Ribozyme insulator	CamR	[1]
pCTK052	2c	RiboJ53	Ribozyme insulator	CamR	[1]
pCTK053	2c	RiboJ54	Ribozyme insulator	CamR	[1]
pCTK054	2c	RiboJ57	Ribozyme insulator	CamR	[1]
pCTK055	2c	RiboJ60	Ribozyme insulator	CamR	[1]
pCTK056	2c	SarJ	Ribozyme insulator	CamR	[1]
pCTK057	2c	ScmJ	Ribozyme insulator	CamR	[1]
pCTK058	2d	B0064	Ribosome binding site	CamR	[1]
pCTK059	2d	RBS-A1	Ribosome binding site	CamR	[1]
pCTK060	2d	RBS-B1	Ribosome binding site	CamR	[1]
pCTK061	2d	RBS-B2	Ribosome binding site	CamR	[1]
pCTK062	2d	RBS-B3	Ribosome binding site	CamR	[1]
pCTK063	2d	RBS-E1	Ribosome binding site	CamR	[1]
pCTK064	2d	RBS-F1	Ribosome binding site	CamR	[1]
pCTK065	2d	RBS-H1	Ribosome binding site	CamR	[1]
pCTK066	2d	RBS-I1	Ribosome binding site	CamR	[1]
pCTK067	2d	RBS-L1	Ribosome binding site	CamR	[1]
pCTK068	2d	RBS-N1	Ribosome binding site	CamR	[1]
pCTK069	2d	RBS-P1	Ribosome binding site	CamR	[1]
pCTK070	2d	RBS-P2	Ribosome binding site	CamR	[1]
pCTK071	2d	RBS-P3	Ribosome binding site	CamR	[1]
pCTK072	2d	RBS-Q1	Ribosome binding site	CamR	[1]
pCTK073	2d	RBS-Q2	Ribosome binding site	CamR	[1]
pCTK074	2d	RBS-R1	Ribosome binding site	CamR	[1]
pCTK075	2d	RBS-S1	Ribosome binding site	CamR	[1]
pCTK076	2d	RBS-S2	Ribosome binding site	CamR	[1]
pCTK077	2d	RBS-S3	Ribosome binding site	CamR	[1]
pCTK078	2d	RBS-S4	Ribosome binding site	CamR	[1]
pCTK079	3	mCherry	Fluorescent protein	CamR	Lab stock
pCTK080	3	sfGFP	Fluorescent protein	CamR	Lab stock
pCTK081	3	yfp	Fluorescent protein	CamR	[1]
pCTK082	3	ameR	Repressor	CamR	[1]
pCTK083	3	amtR	Repressor	CamR	[1]
pCTK084	3	betI	Repressor	CamR	[1]
pCTK085	3	bm3R1	Repressor	CamR	[1]
pCTK086	3	hlyIIR	Repressor	CamR	[1]
pCTK087	3	icaRA	Repressor	CamR	[1]
pCTK088	3	litR	Repressor	CamR	[1]
pCTK089	3	lmrR	Repressor	CamR	[1]
pCTK090	3	phlF	Repressor	CamR	[1]
pCTK091	3	psrA	Repressor	CamR	[1]
pCTK092	3	qacR	Repressor	CamR	[1]
pCTK093	3	srpR	Repressor	CamR	[1]
pCTK094	3	lacI	Repressor	CamR	[1]
pCTK095	3	TetR	Repressor	CamR	[1]
pCTK096	3a	mCherry	Fluorescent protein	CamR	Lab stock
pCTK097	3a	sfGFP	Fluorescent protein	CamR	Lab stock
pCTK098	3a	yfp	Fluorescent protein	CamR	[1]
pCTK099	4	BBa_B0015	Terminator	CamR	[1], [3]
pCTK100	4	ECK120010818	Terminator	CamR	[1]
pCTK101	4	ECK120015170	Terminator	CamR	[1]
pCTK102	4	ECK120015440	Terminator	CamR	[1]
pCTK103	4	ECK120029600	Terminator	CamR	[1]

pCTK104	4	ECK120033736	Terminator	CamR	[1]
pCTK105	4	ECK120033737	Terminator	CamR	[1]
pCTK106	4	L3S2P21	Terminator	CamR	[1]
pCTK107	4	L3S2P24	Terminator	CamR	[1]
pCTK108	4	L3S2P55	Terminator	CamR	[1]
pCTK109	4	L3S3P11	Terminator	CamR	[1]
pCTK110	4	L3S3P31	Terminator	CamR	[1]
pCTK111	4	t500	Terminator	CamR	Lab stock
pCTK112	4b	L3S2P21	Terminator	CamR	[1]
pCTK113	4b	t500	Terminator	CamR	Lab stock
pCTK114	678	p15A ori-AmpR [GFP]	Backbone (GFP dropout)	AmpR	This work
pCTK115	678	p15A ori-AmpR-LacI -TetR [GFP]	Backbone (GFP dropout)	AmpR	This work
pCTK116	678	p15A ori-AmpR-AraC -LacI-TetR [GFP]	Backbone (GFP dropout)	AmpR	This work
pCTK117	678	ColE1 ori-AmpR-AraC -LacI-TetR [GFP]	Backbone (GFP dropout)	AmpR	This work
pCTK118	678	p15A ori-KanR [GFP]	Backbone (GFP dropout)	KanR	This work
pCTK119	678	p15A ori-KanR-LacI -TetR [GFP]	Backbone (GFP dropout)	KanR	This work
pCTK120	678	p15A ori-KanR-AraC -LacI-TetR [GFP]	Backbone (GFP dropout)	KanR	This work
pCTK121	678	pSC101 ori-SmR [GFP]	Backbone (GFP dropout)	SpecR	This work
pCTK122	678	p15A ori-AmpR [RFP]	Backbone (RFP dropout)	AmpR	This work
pCTK123	678	p15A ori-AmpR-LacI -TetR [RFP]	Backbone (RFP dropout)	AmpR	This work
pCTK124	678	p15A ori-AmpR-AraC -LacI-TetR [RFP]	Backbone (RFP dropout)	AmpR	This work
pCTK125	678	ColE1 ori-AmpR-AraC -LacI-TetR [RFP]	Backbone (RFP dropout)	AmpR	This work
pCTK126	678	p15A ori-KanR [RFP]	Backbone (RFP dropout)	KanR	This work
pCTK127	678	p15A ori-KanR-LacI -TetR [RFP]	Backbone (RFP dropout)	KanR	This work
pCTK128	678	p15A ori-KanR-AraC -LacI-TetR [RFP]	Backbone (RFP dropout)	KanR	This work
pCTK129	678	pSC101 ori-SmR [RFP]	Backbone (RFP dropout)	SpecR	This work
pCTK130	2	J23101-RiboJ-B0064	Promoter-ribozyme-RBS	CamR	This work
pCTK131	2	PBAD-RiboJ-B0064	Promoter-ribozyme-RBS	CamR	This work
pCTK132	2	PTet-RiboJ-B0064	Promoter-ribozyme-RBS	CamR	This work
pCTK133	2cd	RiboJ-B0064	Ribozyme-RBS	CamR	This work
pCTK134	2cd34	A1-amtR	NOT gate: BydvJ-A1-amtR-L3S2P55	CamR	[1]
pCTK135	2cd34	B1-bm3R1	NOT gate: SarJ-B1-bm3R1-L3S2P55	CamR	[1]
pCTK136	2cd34	B2-bm3R1	NOT gate: SarJ-B2-bm3R1-L3S2P55	CamR	[1]

pCTK137	2cd34	B3-bm3R1	NOT gate: SarJ-B3-bm3R1-L3S2P55	CamR	[1]
pCTK138	2cd34	E1-betI	NOT gate: RiboJ57-E1-betI-L3S2P55	CamR	[1]
pCTK139	2cd34	F1-ameR	NOT gate: RiboJ54-F1-ameR-L3S2P55	CamR	[1]
pCTK140	2cd34	H1-hlyIIR	NOT gate: RiboJ51-H1-hlyIIR-ECK120033736	CamR	[1]
pCTK141	2cd34	I1-IcaRA	NOT gate: ElvJ-I1-IcaRA-ECK120029600	CamR	[1]
pCTK142	2cd34	L1-LitR	NOT gate: PlmJ-L1-LitR-L3S2P21	CamR	[1]
pCTK143	2cd34	N1-LmrA	NOT gate: RiboJ60-N1-LmrA-L3S2P21	CamR	[1]
pCTK144	2cd34	P1-phlF	NOT gate: RiboJ53-P1-phlF-ECK120033737	CamR	[1]
pCTK145	2cd34	P2-phlF	NOT gate: RiboJ-P2-phlF-ECK120033737	CamR	[1]
pCTK146	2cd34	P3-phlF	NOT gate: RiboJ53-P3-phlF-ECK120033737	CamR	[1]
pCTK147	2cd34	Q1-QacR	NOT gate: RiboJ60-Q1-QacR-L3S2P21	CamR	[1]
pCTK148	2cd34	Q2-QacR	NOT gate: RiboJ60-Q2-QacR-L3S2P21	CamR	[1]
pCTK149	2cd34	R1-PsrA	NOT gate: ScmJ-R1-PsrA-L3S2P21	CamR	[1]
pCTK150	2cd34	S1-srpR	NOT gate: RiboJ10-S1-srpR-ECK120029600	CamR	[1]
pCTK151	2cd34	S2-srpR	NOT gate: RiboJ10-S2-srpR-ECK120029600	CamR	[1]
pCTK152	2cd34	S3-srpR	NOT gate: RiboJ10-S3-srpR-ECK120029600	CamR	[1]
pCTK153	2cd34	S4-srpR	NOT gate: RiboJ10-S4-srpR-ECK120029600	CamR	[1]
pCTK154	2cd34	sfGFP expression	Expression	CamR	This work
pCTK155	2cd34	yfp expression	Expression	CamR	This work
pCTK156	234r	RFP dropout	Dropout part	CamR	This work

Supplementary Table S2: List of plasmids made for characterization results

Note: Plasmids L1-LEU-1, L1-LEU-1E, and L1-LEU-2E are backbone plasmids provided by Maik Molderings (unpublished results) that function as type 56781 parts to make cloning more efficient. These parts are very similar to the cloning backbones of the MYT kit [4] except that they contain isolating ribozymes in the backbone that isolates gene expression between transcriptional units. With the ribozymes in type 2c, this should not be required to achieve good expression results, but doesn't decrease effectiveness either.

Plasmid	Level	Type	Description	Resistance	Components
pJCM113	1	TU1	PTac-A1-amtR	AmpR	L1-LEU-1, pCTK014, pCTK046, pCTK059, pCTK083, pCTK108
pJCM114	1	TU1	PTac-B1-bm3R1	AmpR	L1-LEU-1, pCTK014, pCTK056, pCTK060, pCTK085, pCTK108
pJCM115	1	TU1	PTac-B2-bm3R1	AmpR	L1-LEU-1, pCTK014, pCTK056, pCTK061, pCTK085, pCTK108
pJCM116	1	TU1	PTac-B3-bm3R1	AmpR	L1-LEU-1, pCTK014, pCTK056, pCTK062, pCTK085, pCTK108
pJCM117	1	TU1	PTac-E1-betI	AmpR	L1-LEU-1, pCTK014, pCTK054, pCTK063, pCTK084, pCTK108
pJCM118	1	TU1	PTac-F1-ameR	AmpR	L1-LEU-1, pCTK014, pCTK053, pCTK064, pCTK082, pCTK108
pJCM119	1	TU1	PTac-H1-hlyIIR	AmpR	L1-LEU-1, pCTK014, pCTK051, pCTK065, pCTK086, pCTK104
pJCM120	1	TU1	PTac-I1-IcaRA	AmpR	L1-LEU-1, pCTK014, pCTK047, pCTK066, pCTK087, pCTK103
pJCM121	1	TU1	PTac-L1-LitR	AmpR	L1-LEU-1, pCTK014, pCTK048, pCTK067, pCTK088, pCTK106
pJCM122	1	TU1	PTac-N1-LmrA	AmpR	L1-LEU-1, pCTK014, pCTK055, pCTK068, pCTK089, pCTK106
pJCM123	1	TU1	PTac-P1-phlF	AmpR	L1-LEU-1, pCTK014, pCTK052, pCTK069, pCTK090, pCTK105
pJCM124	1	TU1	PTac-P2-phlF	AmpR	L1-LEU-1, pCTK014, pCTK052, pCTK070, pCTK090, pCTK105
pJCM125	1	TU1	PTac-P3-phlF	AmpR	L1-LEU-1, pCTK014, pCTK052, pCTK071, pCTK090, pCTK105
pJCM126	1	TU1	PTac-Q1-QacR	AmpR	L1-LEU-1, pCTK014, pCTK055, pCTK072, pCTK092, pCTK105
pJCM127	1	TU1	PTac-Q2-QacR	AmpR	L1-LEU-1, pCTK014, pCTK055, pCTK073, pCTK092, pCTK106
pJCM128	1	TU1	PTac-R1-PsrA	AmpR	L1-LEU-1, pCTK014, pCTK057, pCTK074, pCTK091, pCTK106
pJCM129	1	TU1	PTac-S1-srpR	AmpR	L1-LEU-1, pCTK014, pCTK050, pCTK075, pCTK093, pCTK103
pJCM130	1	TU1	PTac-S2-srpR	AmpR	L1-LEU-1, pCTK014, pCTK050, pCTK076, pCTK093, pCTK103
pJCM131	1	TU1	PTac-S3-srpR	AmpR	L1-LEU-1, pCTK014, pCTK050, pCTK077, pCTK093, pCTK103
pJCM132	1	TU1	PTac-S4-srpR	AmpR	L1-LEU-1, pCTK014, pCTK050, pCTK078, pCTK093, pCTK103
pJCM133	1	TU2e	J23101-sfGFP	AmpR	L1-LEU-2E, pCTK029, pCTK049, pCTK058, pCTK080, pCTK106
pJCM135	1	TU2e	PAmeR-sfGFP	AmpR	L1-LEU-2E, pCTK001, pCTK049, pCTK058, pCTK080, pCTK106

pJCM137	1	TU2e	PAmtR-sfGFP	AmpR	L1-LEU-2E, pCTK002, pCTK049, pCTK058, pCTK080, pCTK106
pJCM139	1	TU2e	PBetI-sfGFP	AmpR	L1-LEU-2E, pCTK004, pCTK049, pCTK058, pCTK080, pCTK106
pJCM141	1	TU2e	PBM3R1-sfGFP	AmpR	L1-LEU-2E, pCTK005, pCTK049, pCTK058, pCTK080, pCTK106
pJCM143	1	TU2e	PHlyIIR-sfGFP	AmpR	L1-LEU-2E, pCTK006, pCTK049, pCTK058, pCTK080, pCTK106
pJCM145	1	TU2e	PIcaRA-sfGFP	AmpR	L1-LEU-2E, pCTK007, pCTK049, pCTK058, pCTK080, pCTK106
pJCM147	1	TU2e	PLitR-sfGFP	AmpR	L1-LEU-2E, pCTK008, pCTK049, pCTK058, pCTK080, pCTK106
pJCM149	1	TU2e	PLmrA-sfGFP	AmpR	L1-LEU-2E, pCTK009, pCTK049, pCTK058, pCTK080, pCTK106
pJCM151	1	TU2e	PPhlF-sfGFP	AmpR	L1-LEU-2E, pCTK010, pCTK049, pCTK058, pCTK080, pCTK106
pJCM153	1	TU2e	PPsrA-sfGFP	AmpR	L1-LEU-2E, pCTK011, pCTK049, pCTK058, pCTK080, pCTK106
pJCM155	1	TU2e	PQacR-sfGFP	AmpR	L1-LEU-2E, pCTK012, pCTK049, pCTK058, pCTK080, pCTK106
pJCM157	1	TU2e	SrpR-sfGFP	AmpR	L1-LEU-2E, pCTK013, pCTK049, pCTK058, pCTK080, pCTK106
pJCM212	2		PTac-A1-amtR_PAmtR-sfGFP	KanR	pJCM471, pJCM113, pJCM137
pJCM213	2		PTac-B1-bm3R1_PBM3R1-sfGFP	KanR	pJCM471, pJCM114, pJCM141
pJCM214	2		PTac-B2-bm3R1_PBM3R1-sfGFP	KanR	pJCM471, pJCM115, pJCM141
pJCM215	2		PTac-B3-bm3R1_PBM3R1-sfGFP	KanR	pJCM471, pJCM116, pJCM141
pJCM216	2		PTac-E1-betI_PBetI-sfGFP	KanR	pJCM471, pJCM117, pJCM139
pJCM217	2		PTac-F1-ameR_PAmeR-sfGFP	KanR	pJCM471, pJCM118, pJCM135
pJCM218	2		PTac-H1-hlyIIR_PHlyIIR-sfGFP	KanR	pJCM471, pJCM119, pJCM143
pJCM219	2		PTac-I1-IcaRA_PIcaRA-sfGFP	KanR	pJCM471, pJCM120, pJCM145
pJCM220	2		PTac-L1-LitR_PLitR-sfGFP	KanR	pJCM471, pJCM121, pJCM147
pJCM221	2		PTac-N1-LmrA_PLmrA-sfGFP	KanR	pJCM471, pJCM122, pJCM149
pJCM222	2		PTac-P1-phlF_PPhIF-sfGFP	KanR	pJCM471, pJCM123, pJCM151
pJCM223	2		PTac-P2-phlF_PPhIF-sfGFP	KanR	pJCM471, pJCM124, pJCM151

pJCM224	2		PTac-P3-phlF_PPhIF-sfGFP	KanR	pJCM471, pJCM125, pJCM151
pJCM225	2		PTac-Q1-QacR_PQacR-sfGFP	KanR	pJCM471, pJCM126, pJCM155
pJCM226	2		PTac-Q2-QacR_PQacR-sfGFP	KanR	pJCM471, pJCM127, pJCM155
pJCM227	2		PTac-R1-PsrA_PPsrA-sfGFP	KanR	pJCM471, pJCM128, pJCM153
pJCM228	2		PTac-S1-srpR_PSrpR-sfGFP	KanR	pJCM471, pJCM129, pJCM157
pJCM229	2		PTac-S2-srpR_PSrpR-sfGFP	KanR	pJCM471, pJCM130, pJCM157
pJCM230	2		PTac-S3-srpR_PSrpR-sfGFP	KanR	pJCM471, pJCM131, pJCM157
pJCM231	2		PTac-S4-srpR_PSrpR-sfGFP	KanR	pJCM471, pJCM132, pJCM157
pJCM386	1	TU1e	PJ23101-sfGFP	AmpR	L1-LEU-1E, pCTK029, pCTK154
pJCM387	1	TU1e	PTac-sfGFP	AmpR	L1-LEU-1E, pCTK014, pCTK154
pJCM434	2		PJ23101-sfGFP	KanR	pJCM471, pJCM386
pJCM435	2		PTac-sfGFP	KanR	pJCM471, pJCM387
pJCM446	1	TU1e	PBAD-sfGFP	AmpR	L1-LEU-1E, pCTK003, pCTK154
pJCM447	1	TU1e	PTet-sfGFP	AmpR	L1-LEU-1E, pCTK015, pCTK154
pJCM448	2		PBAD-sfGFP	KanR	pJCM471, pJCM446
pJCM449	2		PTet-sfGFP	KanR	pJCM471, pJCM447
pJCM471	1	BB	p15A ori KanR AraC-LacI-TetR	KanR	pCTK120. pYTK008, pCTK156, pYTK073
pJCM534	1	TU1e	PJ23102-sfGFP	AmpR	L1-LEU-1E, pCTK030, pCTK154
pJCM535	1	TU1e	PJ23103-sfGFP	AmpR	L1-LEU-1E, pCTK031, pCTK154
pJCM536	1	TU1e	PJ23104-sfGFP	AmpR	L1-LEU-1E, pCTK032, pCTK154
pJCM537	1	TU1e	PJ23105-sfGFP	AmpR	L1-LEU-1E, pCTK033, pCTK154
pJCM538	1	TU1e	PJ23106-sfGFP	AmpR	L1-LEU-1E, pCTK034, pCTK154
pJCM539	1	TU1e	PJ23107-sfGFP	AmpR	L1-LEU-1E, pCTK035, pCTK154
pJCM540	1	TU1e	PJ23108-sfGFP	AmpR	L1-LEU-1E, pCTK036, pCTK154
pJCM541	1	TU1e	PJ23109-sfGFP	AmpR	L1-LEU-1E, pCTK037, pCTK154
pJCM542	1	TU1e	PJ23110-sfGFP	AmpR	L1-LEU-1E, pCTK038, pCTK154
pJCM543	1	TU1e	PJ23111-sfGFP	AmpR	L1-LEU-1E, pCTK039, pCTK154
pJCM544	1	TU1e	PJ23113-sfGFP	AmpR	L1-LEU-1E, pCTK040, pCTK154
pJCM545	1	TU1e	PJ23115-sfGFP	AmpR	L1-LEU-1E, pCTK041, pCTK154
pJCM546	1	TU1e	PJ23116-sfGFP	AmpR	L1-LEU-1E, pCTK042, pCTK154
pJCM547	1	TU1e	PJ23117-sfGFP	AmpR	L1-LEU-1E, pCTK043, pCTK154
pJCM548	1	TU1e	PJ23118-sfGFP	AmpR	L1-LEU-1E, pCTK044, pCTK154
pJCM549	1	TU1e	PJ23119-sfGFP	AmpR	L1-LEU-1E, pCTK045, pCTK154
pJCM550	2		PJ23102-sfGFP	KanR	pJCM471, pJCM534
pJCM551	2		PJ23103-sfGFP	KanR	pJCM471, pJCM535
pJCM552	2		PJ23104-sfGFP	KanR	pJCM471, pJCM536
pJCM553	2		PJ23105-sfGFP	KanR	pJCM471, pJCM537
pJCM554	2		PJ23106-sfGFP	KanR	pJCM471, pJCM538
pJCM555	2		PJ23107-sfGFP	KanR	pJCM471, pJCM539
pJCM556	2		PJ23108-sfGFP	KanR	pJCM471, pJCM540
pJCM557	2		PJ23109-sfGFP	KanR	pJCM471, pJCM541
pJCM558	2		PJ23110-sfGFP	KanR	pJCM471, pJCM542
pJCM559	2		PJ23111-sfGFP	KanR	pJCM471, pJCM543
pJCM560	2		PJ23113-sfGFP	KanR	pJCM471, pJCM544
pJCM561	2		PJ23115-sfGFP	KanR	pJCM471, pJCM545
pJCM562	2		PJ23116-sfGFP	KanR	pJCM471, pJCM546
pJCM563	2		PJ23117-sfGFP	KanR	pJCM471, pJCM547
pJCM564	2		PJ23118-sfGFP	KanR	pJCM471, pJCM548
pJCM565	2		PJ23119-sfGFP	KanR	pJCM471, pJCM549

Supplementary Table S3: Constitutive promoter expression levels.

**Promoter**	**CTK Characterization (RPU)**	**Anderson characterization (RPU)**[2]*
J23119	7.76	N/A
J23111	3.97	0.83
J23104	1.64	1.03
J23101	1.00	1.00
J23102	0.98	1.23
J23118	0.75	0.80
J23107	0.46	0.51
J23110	0.41	0.47
J23106	0.37	0.67
J23113	0.27	0.01
J23108	0.27	0.73
J23105	0.27	0.34
J23103	0.27	0.01
J23117	0.22	0.09
J23109	0.16	0.06
J23116	0.15	0.23
J23115	0.08	0.21

*The RPU of the Anderson characterization was converted from normalization to J23100 to normalization to J23101.

Supplementary Table S4: Inducible promoter dose-response parameters

Plasmid	Promoter	Y_max	Y_min	n	K	Dynamic range
pJCM435	PTac	2.794	0.014	1.813	256.617	194.464
pJCM448	PBAD	1.084	0.015	2.24	633.467	73.388
pJCM449	PTet	8.582	0.033	2.117	10.119	263.919

Supplementary Table S5: NOT gate response function parameters

Plasmid	Gate	Y_max	Y_min	n	K	Dynamic range
pJCM212	A1-AmtR	5.49	0.043	1.863	0.011	128.658
pJCM213	B1-BM3R1	0.479	0.005	2.378	0.01	105.956
pJCM214	B2-BM3R1	0.335	0.025	1.442	0.072	13.622
pJCM215	B3-BM3R1	0.383	0.094	0.846	0.843	4.087
pJCM216	E1-BetI	9.191	0.121	2.081	0.066	75.669
pJCM217	F1-AmeR	5.374	0.431	1.323	0.076	12.46
pJCM218	H1-HlyIIR	5.049	0.098	1.7	0.086	51.392
pJCM219	I1-IcaRA	5.029	0.098	2.575	0.03	51.362
pJCM220	L1-LitR	0.36	0.01	1.262	0.025	37.681
pJCM221	N1-LmrA	1.211	0.021	1.429	0.036	56.789
pJCM222	P1-PhlF	7.194	0.048	3.159	0.039	150.738
pJCM223	P2-PhlF	7.589	0.05	2.222	0.103	152.48
pJCM224	P3-PhlF	8.274	0.058	2.741	0.047	143.35
pJCM225	Q1-QacR	8.525	0.047	2.534	0.023	181.924
pJCM226	Q2-QacR	8.107	0.046	1.601	0.057	178.172
pJCM227	R1-PsrA	7.551	0.173	1.086	0.073	43.765
pJCM228	S1-SrpR	0.957	0.005	3.971	0.011	195.516
pJCM229	S2-SrpR	1.837	0.011	2.558	0.028	160.211
pJCM230	S3-SrpR	1.987	0.012	1.721	0.047	160.701
pJCM231	S4-SrpR	1.772	0.01	2.18	0.051	170.457

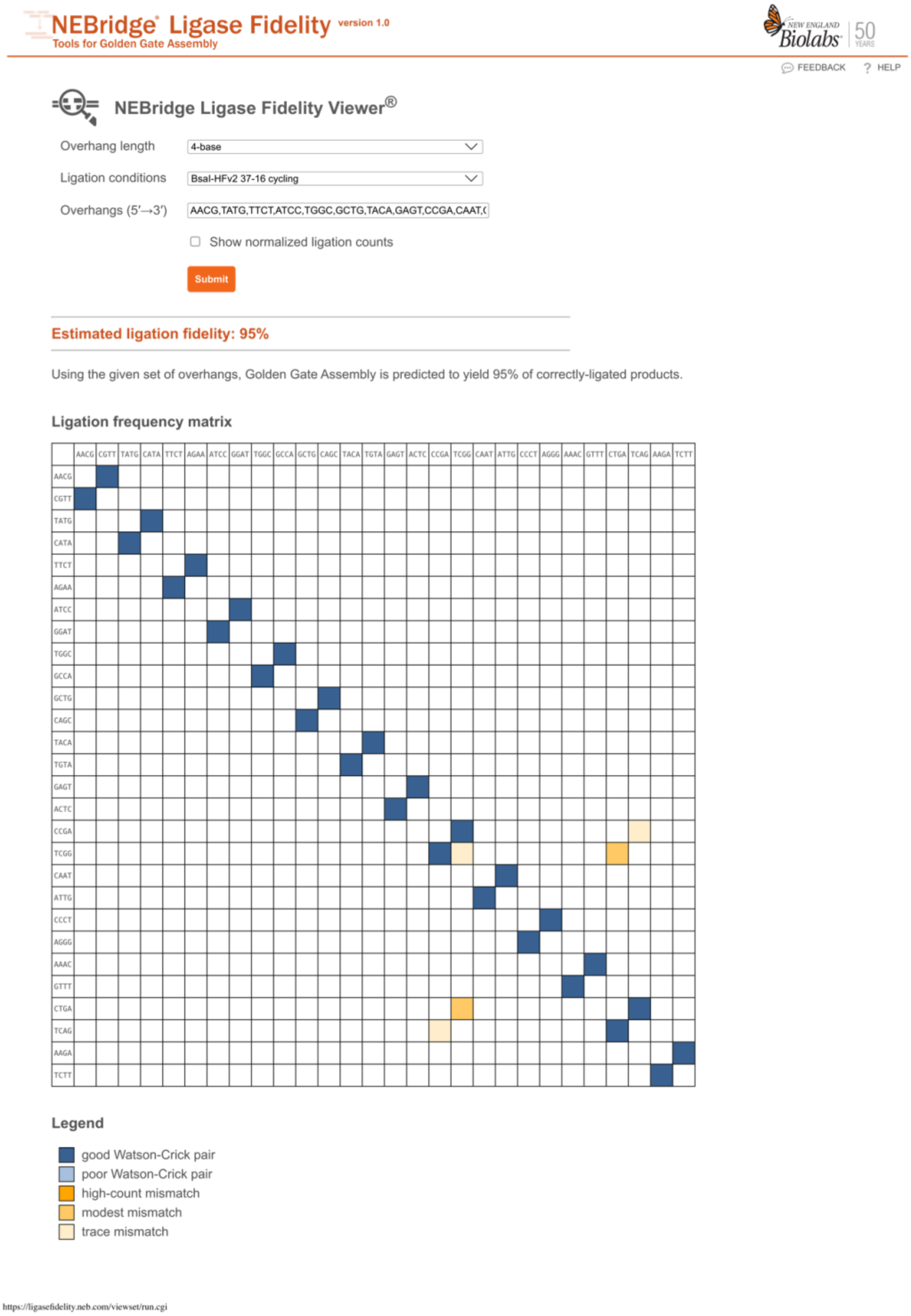

Supplementary Figure S1: Ligase fidelity overview of the 4-base pair overhangs employed in the CTK. The additional overhangs, **AAAC** (between 2a and 2b), **CTGA** (between 2b and 2c), and **AAGA** (between 2c and 2d), show minimal overlap with the existing YTK overhangs. The only noticeable mismatches are between CTGA and TCGG. These are the overhangs between Type 2b and 2c and between Type 7 and 8 parts. When Type 678 backbones are used for construction of level 1 plasmids, this potential mismatch is entirely circumvented.

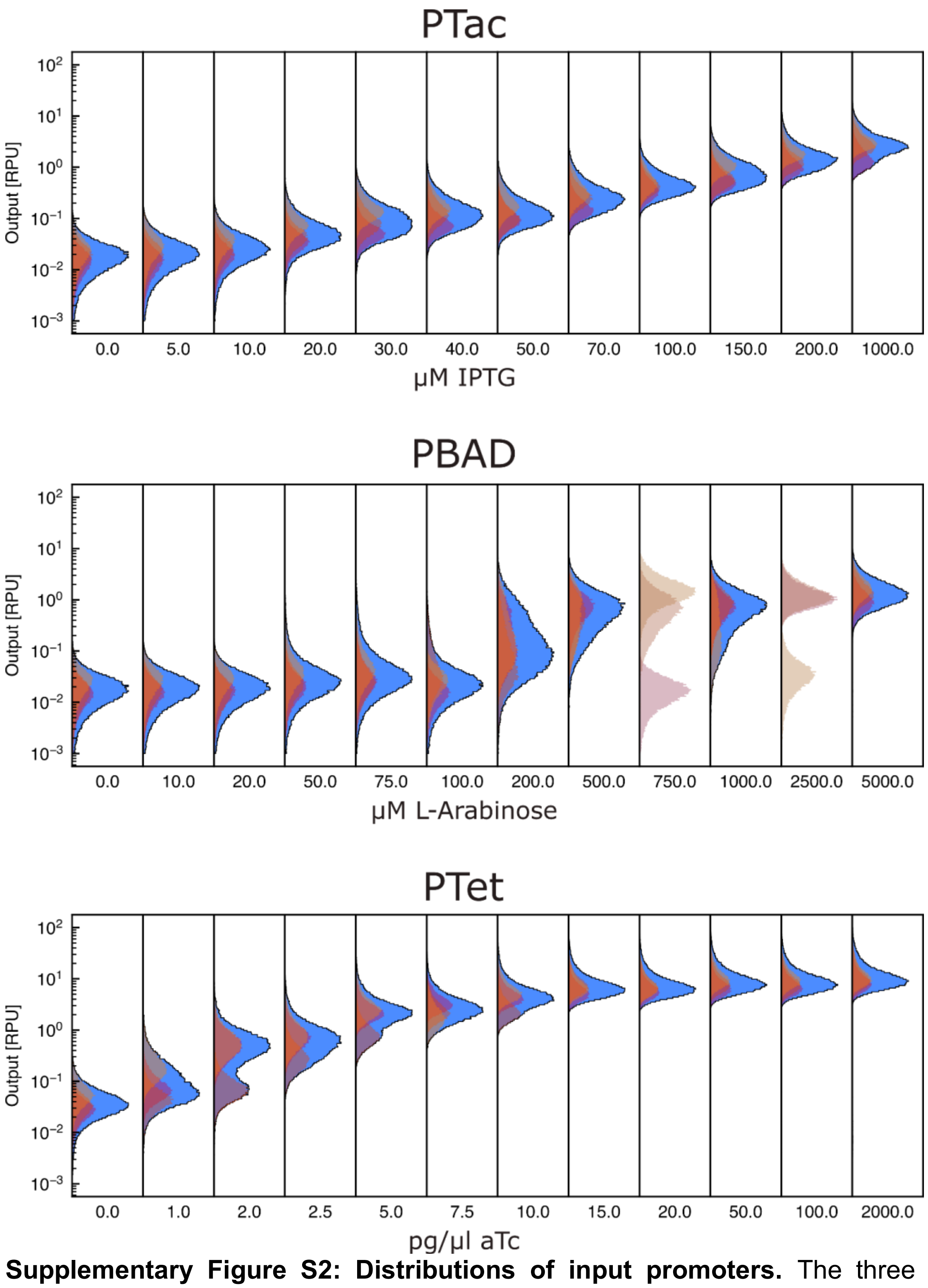

Supplementary Figure S2: Distributions of input promoters. The three input promoters were measured at 12 different concentrations of their respective chemical inducers. For each measurement, three replicates were performed on separate days. The individual replicates can be seen in yellow, orange and pink. The larger peak in light blue is the merged population of the replicates. Populations in light colors were outliers and not included in further calculations (see Methods). Parameters for a dose-response curve fit based on these distributions can be seen in Supplementary Table S4.

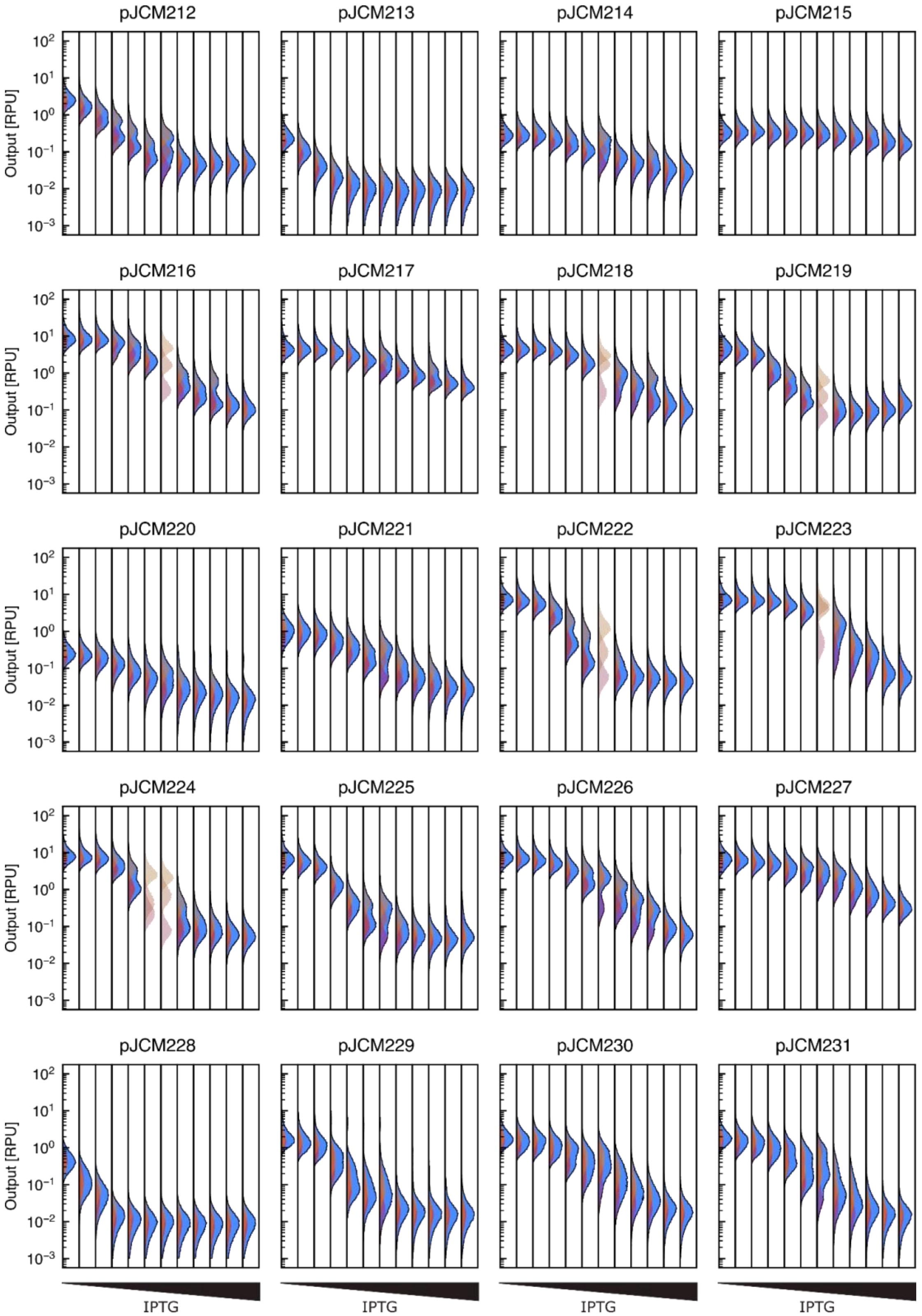

Supplementary Figure S3: Distributions of NOT gates. The distributions of the 20 NOT gates, each normalized to RPU. For each measurement, three replicates were performed on separate days. The individual replicates can be seen in yellow, orange and pink. The larger peak in light blue is the merged population of the replicates. Populations in light colors were outliers and not included in further calculations (see Methods). IPTG concentrations used in this experiment were (from left to right): 0, 5, 10, 20, 30, 40, 50, 70, 100, 150, 200, and 1000 µM.

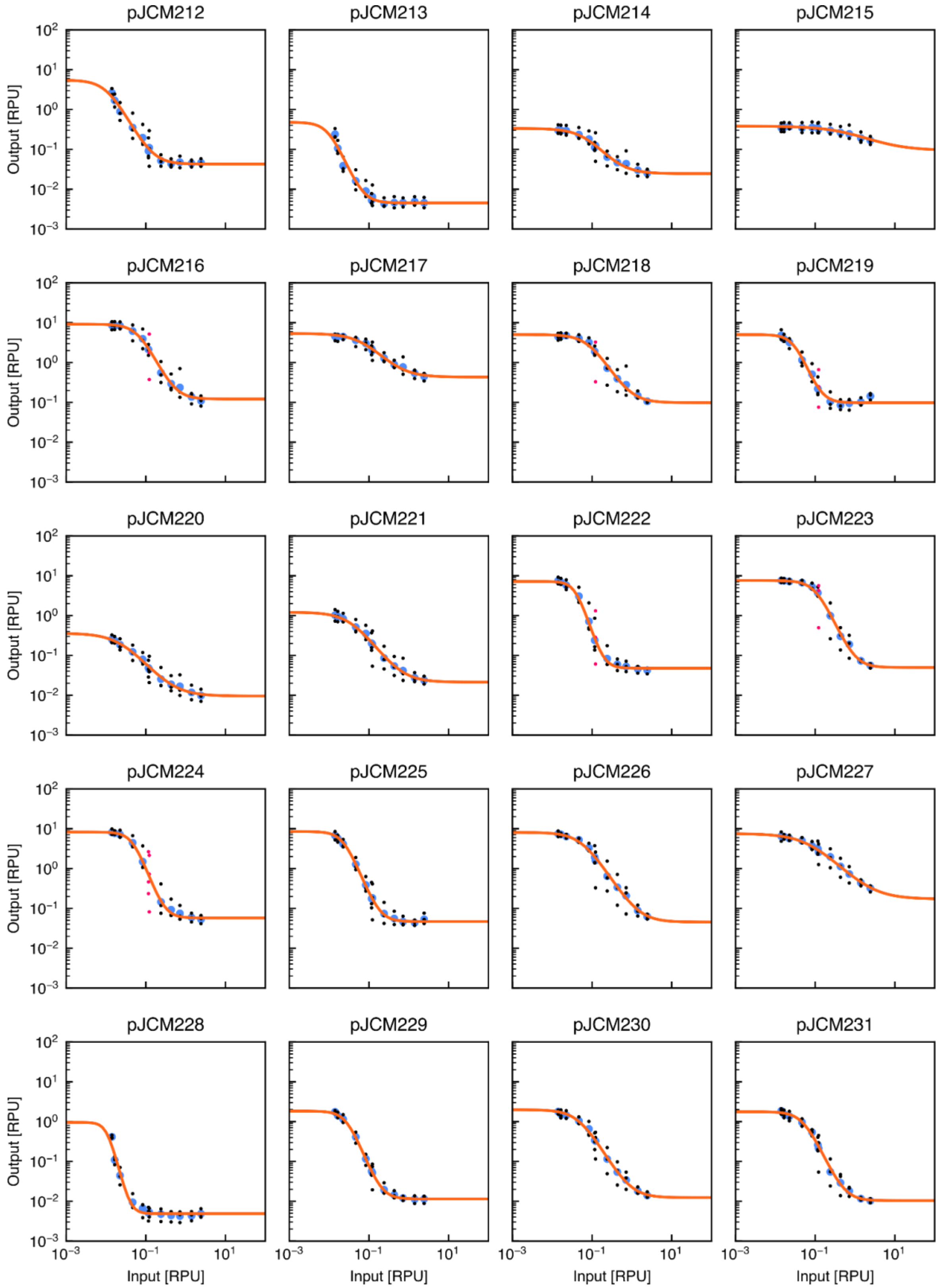

Supplementary Figure S4: Response functions of NOT gates. The fitted response function of the 20 NOT gates, each normalized to RPU on both input and output. The black points are individual measurements from the three replicates and the blue points are the median of the merged replicates. Points in red were outliers and not included in the merged populations. Parameters of the fitted curves can be seen in Supplementary Table S5.

Supplementary Text: Benchmarking clustering algorithm

For benchmarking, we used three different approaches: First, a naive approach where random nucleotides are added until the fragment is at least 300 bases long. This method will provide the baseline for comparisons between other methods, as it presents the least optimal method of packaging for *de novo* synthesis. The second approach is to randomly group fragments shorter than 300 bases together. Since this method is stochastic, the computations are repeated 10 times and both the best and the average results are reported. Third, we investigate using clustering to identify good groupings that will minimize synthesis errors while maximizing compactness.

To benchmark this tool, we chose 3 sets of DNA parts that contain many smaller parts to highlight the efficiency of the software. For all DNA sets, CDSs were excluded from this benchmarking, since they are above 300 bases and therefore would add equally to all methods tested.

The first set are the parts contained within the CTK. This set has a total of 108 parts, which breaks down into 45 promoters, 12 ribozymes, 21 RBSs, 15 CDSs, and 15 terminators.

The second set comes from the paper "Partitioning of a 2-bit hash function across 66 communicating cells" from the Voigt lab [5], was published online in the fall of 2024, and in it, the authors introduce a total of 1.1 Mb of recombinant DNA into *E. coli*. Cloning plasmids for projects of this size can be facilitated by using a hierarchical cloning scheme, such as MoClo. To get these parts into the system, many would have to be *de novo* synthesized. To create all circuits from the paper, a total of 115 parts will be needed. This breaks down into 26 promoters, 12 ribozymes, 33 RBSs, 25 CDSs, and 19 terminators.

The third set is a collection of 15 synthetic *E. coli* promoters that have a very close sequence similarity. This set was chosen as a test case to demonstrate the ability of the software to make even very similar sequences ready for synthesis.

The results of the benchmarks can be seen in the tables below. For all cases, the naive approach is by far the most expensive when compared to any other approach, and it is therefore not recommended.

When comparing the other methods described above, we see that the lowest price can be achieved by using the random clustering for two out of the three datasets. However, for the third set with the 15 promoter variants, the high sequence similarity meant that the random assignment couldn't generate a version that could be synthesized.

Using the random method for the two other DNA sets, 4 out of 10 failed for the CTK parts, and 3 out of 10 failed for the second. Here a failure is defined as having at least one sequence that is not able to be synthesized.

Using the clustering method from our tool, we were able to reduce the synthesis price to 46%, 73%, and 60%, respectively, of the naive approach for the three datasets. Furthermore, in the second set, the aggressive clustering method could decrease the synthesis price even further to 56% of the original naive approach.

Supplementary Table S5: Benchmark of set 1, the CTK parts

Method	Number of fragments	Length (bp)	Price (USD)	Price compared to Naive	Success rate
Naive	108	38282	$3968.16	100%	100% (1/1)
Aggressive clustering	47	20536	$1833.16	46%	100% (1/1)
Clustering	47	20536	$1833.16	46%	100% (1/1)
Average Random	47	20684	$1841.50	46%	60% (6/10)
Best Random	47	20653	$1833.16	46%	

Supplementary Table S6: Benchmark of set 2, the parts required for the 2-bit hashing function

Method	Number of fragments	Length (bp)	Price (USD)	Price compared to Naive	Success rate
Naive	115	46998	4573.10	100%	100% (1/1)
Aggressive clustering	57	31279	2543.10	56%	100% (1/1)
Clustering	63	33099	2753.10	60%	100% (1/1)
Average Random	55	30530	2474.43	54%	70% (7/10)
Best Random	55	30412	2473.10	54%	

Supplementary Table S7: Benchmark of set 3, RcsAB promoters

Method	Number of fragments	Length (bp)	Price (USD)	Price compared to Naive	Success rate
Naive	15	4505	525	100%	100% (1/1)
Aggressive clustering	-	-	-	-	0% (0/1)

Clustering	11	3320	385	73%	100% (1/1)
Average Random	-	-	-	-	0% (0/10)
Best Random	-	-	-	-	

Supplementary References

[1] A. A. K. Nielsen *et al.*, ‘Genetic circuit design automation’, *Science*, vol. 352, no. 6281, p. aac7341, Apr. 2016, doi: 10.1126/science.aac7341.

[2] ‘Promoters/Catalog/Anderson - parts.igem.org’. Accessed: Mar. 17, 2025. [Online]. Available: https://parts.igem.org/Promoters/Catalog/Anderson

[3] ‘Part:BBa B0015 - parts.igem.org’. Accessed: Oct. 07, 2025. [Online]. Available: https://parts.igem.org/Part:BBa_B0015

[4] W. M. Shaw, A. S. Khalil, and T. Ellis, ‘A Multiplex MoClo Toolkit for Extensive and Flexible Engineering of *Saccharomyces cerevisiae*’, *ACS Synth. Biol.*, vol. 12, no. 11, pp. 3393–3405, Nov. 2023, doi: 10.1021/acssynbio.3c00423.

[5] J. P. Padmakumar *et al.*, ‘Partitioning of a 2-bit hash function across 66 communicating cells’, *Nat. Chem. Biol.*, vol. 21, no. 2, pp. 268–279, May 2025, doi: 10.1038/s41589-024-01730-1.
